# Genome Sequence of *Martelella* sp. Strain AD-3, a Moderately Halophilic Polycyclic Aromatic Hydrocarbon-Degrading Bacterium

**DOI:** 10.1128/genomeA.01189-13

**Published:** 2014-01-16

**Authors:** Changzheng Cui, Pengpeng Li, Gao Liu, Hongzhi Tang, Kuangfei Lin, Qishi Luo, Shanshan Liu, Ping Xu, Yongdi Liu

**Affiliations:** State Environmental Protection Key Laboratory of Environmental Risk Assessment and Control on Chemical Process, School of Resources and Environmental Engineering, East China University of Science and Technology, Shanghai, Chinaa; State Key Laboratory of Microbial Metabolism and School of Life Sciences & Biotechnology, Shanghai Jiao Tong University, Shanghai, Chinab; State Environmental Protection Engineering Center for Urban Soil Contamination Control and Remediation, Institute of Wastes and Soil Environment, Shanghai Academy of Environmental Science, Shanghai, Chinac; School of Environmental Science and Engineering, Shanghai Jiao Tong University, Shanghai, Chinad

## Abstract

*Martelella* sp. strain AD-3, enriched from a petroleum-contaminated site with high salinity, can efficiently degrade polycyclic aromatic hydrocarbons. Here, we report the 4.75-Mb genome sequence of strain AD-3 with its genetic feature of helping to remediate environmental organic pollutants.

## GENOME ANNOUNCEMENT

The petroleum industry generates a huge amount of oily and saline wastewater after separation of crude oil from the disposed reservoir. The main contaminants in this water are aromatic and polycyclic aromatic hydrocarbon (PAH) compounds (1, 2). Halophilic bacteria are helpful for removing organic pollutants from high-salinity industrial wastewaters without first reducing the levels of salts (3). *Martelella* sp. strain AD-3 (CCTCC M2011218), a moderate halophilic bacterium, was isolated from a petroleum-contaminated soil with high salinity in China (4). It has high capabilities for degrading some PAHs, such as phenanthrene and anthracene, and it uses the toxicants as its sole carbon source (5). In addition, the strain can tolerate a broad range of salinities (0.1 to 15%) and a range of pHs (6.0 to 10.0) in the process of degrading PAHs (4, 5). To date, few genomes of *Martelella* spp. have been sequenced; thus, this genome report of strain AD-3 may provide new genetic information regarding its remediation of environmental organic toxicants under high-salinity conditions.

Here, we present the draft genome sequence of *Martelella* sp. strain AD-3, determined by using the Illumina HiSeq 2000 system, which was performed with a paired-end library at the Chinese National Human Genome Center, Shanghai, China. The reads of strain AD-3 were assembled into 197 contigs (longest contig size is 215,158 bp, median sequence size is 24,130 bp) using the Velvet software (version 1.2.10) (6). Gene prediction and genome annotation were carried out using RAST (7). The genome sequence of strain AD-3 is composed of 4,753,609 bases with a G+C content of 62.3%. There are 4,496 predicted coding sequences (CDSs), together with 50 RNAs. About 71 CDSs involved in the metabolism of aromatic compounds were predicted.

Genes related to degrading salicylic acid, gentisic acid, *p*-hydroxybenzoate, chloroaromatic compounds, and biphenyl have been annotated in the genome sequence of strain AD-3. These genes or gene clusters will further explain its ability to degrade those organic toxicants. Moreover, there are 68 CDSs, which have been annotated as antibiotics and toxic compound resistance genes, and 242 CDSs annotated for membrane transport. These genes will contribute to the ability of strain AD-3 to remediate a petroleum-contaminated site with high salinity.

### Nucleotide sequence accession numbers.

This whole-genome shotgun project has been deposited at DDBJ/EMBL/GenBank under the accession no. AYGY00000000. The version described in this paper is version AYGY02000000.
